# Nitride oxide synthase 3 and klotho gene polymorphisms in the pathogenesis of chronic kidney disease and age-related cognitive impairment: a systematic review and meta-analysis

**DOI:** 10.12688/f1000research.22989.2

**Published:** 2021-03-19

**Authors:** Atma Gunawan, Jonny Karunia Fajar, Fredo Tamara, Aditya Indra Mahendra, Muhammad Ilmawan, Yeni Purnamasari, Dessy Aprilia Kartini, Eden Suryoiman Winoto, Efriko Septananda Saifillah, Dewi Sri Wulandari, Pratista Adi Krisna, Ema Dianita Mayasari, Tri Wahyudi Iman Dantara, Ramadi Satryo Wicaksono, Djoko Wahono Soeatmadji

**Affiliations:** 1 Division of Nephrology and Hypertension, Department of Internal Medicine, Faculty of Medicine, Universitas Brawijaya, Malang, East Java, 65145, Indonesia; 2 Brawijaya Internal Medicine Research Center, Department of Internal Medicine, Faculty of Medicine, Universitas Brawijaya, Malang, East Java, 65145, Indonesia; 3 Faculty of Medicine, Universitas Brawijaya, Malang, East Java, 65145, Indonesia; 4 Department of Internal Medicine, Rumah Sakit Umum Daerah Bangil., Pasuruan, East Java, 67153, Indonesia; 5 Division of Endocrinology and Metabolic Diseases, Department of Internal Medicine, Faculty of Medicine, Universitas Brawijaya, Malang, East Java, 65145, Indonesia

**Keywords:** Nitride oxide synthase, klotho, chronic kidney disease, age-related cognitive impairment

## Abstract

**Background:** While it has been known that the development of chronic kidney disease (CKD) and age-related cognitive impairment involves several mediators, the evidence in clinical practice only reveals nitride oxide synthase (NOS) and klotho. However, the evidence for this topic is conflicted. The aim of this study was to assess the role of NOS and klotho single nucleotide polymorphisms (SNPs) in the pathogenesis of CKD and age-related cognitive impairment.

**Methods:** We performed a meta-analysis during October to December 2019. Paper collection was performed in major scientific websites, and we extracted information of interest from each paper. Data were analyzed using a Z-test with either random or fixed effect model.

**Results:** Our initial assessment identified *NOS3* G894T, *NOS3* T786C, *NOS3* 4b/4a, klotho ( *KL*) G395A, and *KL* C1818T as the gene candidate for our meta-analysis. Our pooled calculation revealed that *NOS3* G894T was associated with the risk of both age-related cognitive impairment and CKD. Increased susceptibility to age-related cognitive impairment was observed in the GG genotype, and increased risk of CKD was found in patients with a single T allele and TT genotype for *NOS3 *nucleotide 894. For *NOS3* 4b/4a, increased risk of CKD was only found in 4a4a genotype. For *NOS3* T786C, we failed to show the association with both CKD and age-related cognitive impairment. Subsequently, for *KL* G395A, A allele and GA genotype were found to correlate with increased susceptibility to CKD, while its correlation to age-related cognitive impairment was failed to clarify. For *KL* C1818T, our analysis failed to find the correlation with the risk of CKD.

**Conclusions:** Our results reveal that the *NOS3* G894T gene polymorphism has a crucial role in the pathogenesis of both CKD and age-related cognitive impairment.

## Introduction

Aging had remained a challenging topic since the last three decades ^1^. Some large scale studies have been developed to clarify the precise mechanism of how aging affects the body ^2, 3^ and also how to prevent this circumstance ^4^. This is because aging is a normal condition in human life. This means that this proccess has to occur in everyone. Recently, to avoid this circumstance, studies have concerned to elucidate aging because aging is known to correlate with age-related diseases including cardiovascular disease ^5^, stroke ^6^, dementia ^7^, and chronic kidney disease (CKD) ^8^. Of these, CKD was considered the more serious disease because it was proven to associate with high risk of mortality and poor quality of life ^9^. It is widely known that patients with stage V CKD should be treated with regular dialysis and or even renal transplantation ^10^.

CKD is a fatal disease for most populations ^11^. The investigation regarding the better treatment option for this disease had not provided significant development in developing countries. In the context of aging, this might involve several mediators, including estrogen, androgen, L-arginine, nitride oxide synthase (NOS), and klotho ^12^. Of these, only NOS and klotho have been well reported in genetic levels and in clinical settings in the context of aging and CKD. Other mediators, during this time, were only proposed as theory or hypothesis. The absense of direct clinical investigation regarding those mediators in the context of aging and CKD led to these mediators being considered as correlated with aging and CKD. The lack of studies investigating aging in clinical practice might be due to the fact that the definition of aging is complex, and it can be difficult to determine the appropriate scope of aging. However, aging is widely to correlate with age-related cognitive impairment ^13^. For this reason, in our present study, our investigation only concerned age-related cognitive impairment. Furthermore, investigating the role of NOS and klotho gene polymorphisms in the case of age-related cognitive impairment and CKD was logical and crucial for better understanding concerning the development of aging and CKD. Moreover, due to conflicting reports regarding this topic, a meta-analysis study was required to elucidate the real association.

Our current study, therefore, aimed to perform a meta-analysis concerning the role of NOS and klotho gene polymorphisms in the case of age-related cognitive impairment and CKD. Our present study might provide better understanding on which allele or genotype of NOS and klotho gene polymorphisms are associated with the risk of age-related cognitive impairment and CKD.

## Methods

### Study design

During the study time frame (October-December 2019), a meta-analysis was conducted to assess the correlation between NOS and klotho gene polymorphisms and the risk of CKD and age-related cognitive impairment. To attain our purpose, we collected papers from PubMed, Embase, Cochrane, and Web of Science. Moreover, to determine the association and effect estimates, data on allele and genotype frequency from selected papers were used to calculate the odds ratio (OR) and 95% confidence interval (95%CI). The protocols in our current study include paper selection, data extraction, quality assessment, and statistical analysis referred to our previous studies ^14– 18^, and we also used the checklist of the Preferred Reporting Items for Systematic Review and Meta-Analysis (PRISMA) to guide the protocols in our study ^19^. A completed PRISMA checklist for the current study is available (DOI: https://doi.org/10.6084/m9.figshare.12016782) ^20^.

### Eligibility criteria

To obtain the papers, the following criteria should be met to include the papers in our study: (1) assessing the correlation between NOS and klotho gene variants and the risk of CKD and age-related cognitive impairment; and (2) providing sufficient data for calculation of OR and 95%CI. Furthermore, we excluded the papers if the following reasons were met: (1) irrelevant topic, (2) review, (3) non-standard data presentation, (4) deviation from Hardy-Weinberg equilibrium, and (5) double publication. We managed reference list using EndNote v8 (Thompson Reuters, Eagan, Minnesota) to remove instances of double publication.

### Search strategy and data extraction

Papers assessing the association between NOS and klotho gene polymorphisms and the risk of CKD and age-related cognitive impairment were searched in major scientific websites (PubMed, Embase, Cochrane, and Web of Science) up to 5 December 2019. In searching the articles, we restricted the publication language to English. Moreover, to perform a holistic searching, we applied the keywords adapted from medical subject headings (MeSH): ["chronic kidney disease" or "chronic renal failure"] and ["aging" or "age-related cognitive impairment"] and ["nitride oxide synthase" or "NOS"] and ["klotho"]. If we found double publication data, we only included article with the larger sample size. Subsequently, for data extraction, the following information of interest was extracted: (1) first author name; (2) publication year; (3) sample size of case and control, and (4) genotype frequencies of case and control groups.

### Assessment of the methodology quality

To assess the quality of each study, a Methodological Index For Non-Randomized Studies (MINORS) scoring system was applied ^21^. The MINORS score ranged from 0 to 24 and consisted of 12 items. Each item was assessed as 0 if the item was not reported, 1 if the item was inadequate reported, and 2 if the item was adequate reported. Each study was interpreted as having low quality if the score was less than or equal to 12, moderate if the score was less than or equal to 16 and more than 12, and high quality if the score was more than 16 ^21^.

### Outcome measure

Our initial searching identified five single nucleotide polymorphisms (SNPs) for included in our meta-analysis: *NOS3* 4b/4a, *NOS3* G894T, *NOS3* T786C, klotho ( *KL*) G395A, and *KL* C1818T. For age-related cognitive impairment, the SNPs were *NOS3* G894T, *NOS3* T786C, and *KL* G395A. For CKD, the SNPs were *NOS3* 4b4a, *NOS3* G894T, *NOS3* T786C, *KL* G395A, and *KL* C1818T. In each SNP, data analysis was performed in all alleles and genotypes models to assess the correlation and effect estimates. For *NOS3* 4b/4a, the genetic models were 4b vs. 4a; 4a vs. 4b; 4b4b vs. 4b4a+4a4a; 4b4a vs. 4b4b+4a4a; and 4a4a vs. 4b4b+4b4a. For *NOS3* G894T, the genetic models were G vs. T; T vs. G; GG vs. GT+TT; GT vs. GG+TT; and TT vs. GG+GT. For *NOS3* T786C, the genetic models were T vs. C; C vs. T; TT vs. TC+CC; TC vs. TT+CC; and CC vs. TT+TC. For *KL* G395A, the genetic models were as follows: G vs. A; A vs. G; GG vs. GA+AA; GA vs. GG+AA; and AA vs. GG+GA. For *KL* C1818T, the genetic models were C vs. T; T vs. C; CC vs. CT+TT; CT vs. CC+TT; and TT vs. CC+CT.

### Statistical analysis

The association and effect estimation between *NOS3* and *KL* gene variants and the risk of CKD and age-related cognitive impairment were determined using a Z-test. The p value of less than 0.05 was considered statistically significant. Moreover, to determine effect estimates, the calculation of pooled OR and 95%CI was performed. Prior to determining the association and effect estimation, to assess the consistency in our meta-analysis, data were analyzed for heterogeneity and potential publication bias. For assessing the heterogeneity, we applied a Q-test. A p-value of less than 0.10 was considered to indicate heterogeneity and data were analyzed using random effect model. Conversely, we used fixed effect model if the p value was more than 0.01. Moreover, for testing the potential for publication bias, Egger's test was employed. A p-value of less than 0.05 was considered as indicating publication bias. All analyses in our present study were performed using Review Manager [Revman Cochrane, London, UK] version 5.3. The cummulative calculation was presented using a forest plot.

## Results

### Paper selection

**Figure 1.**
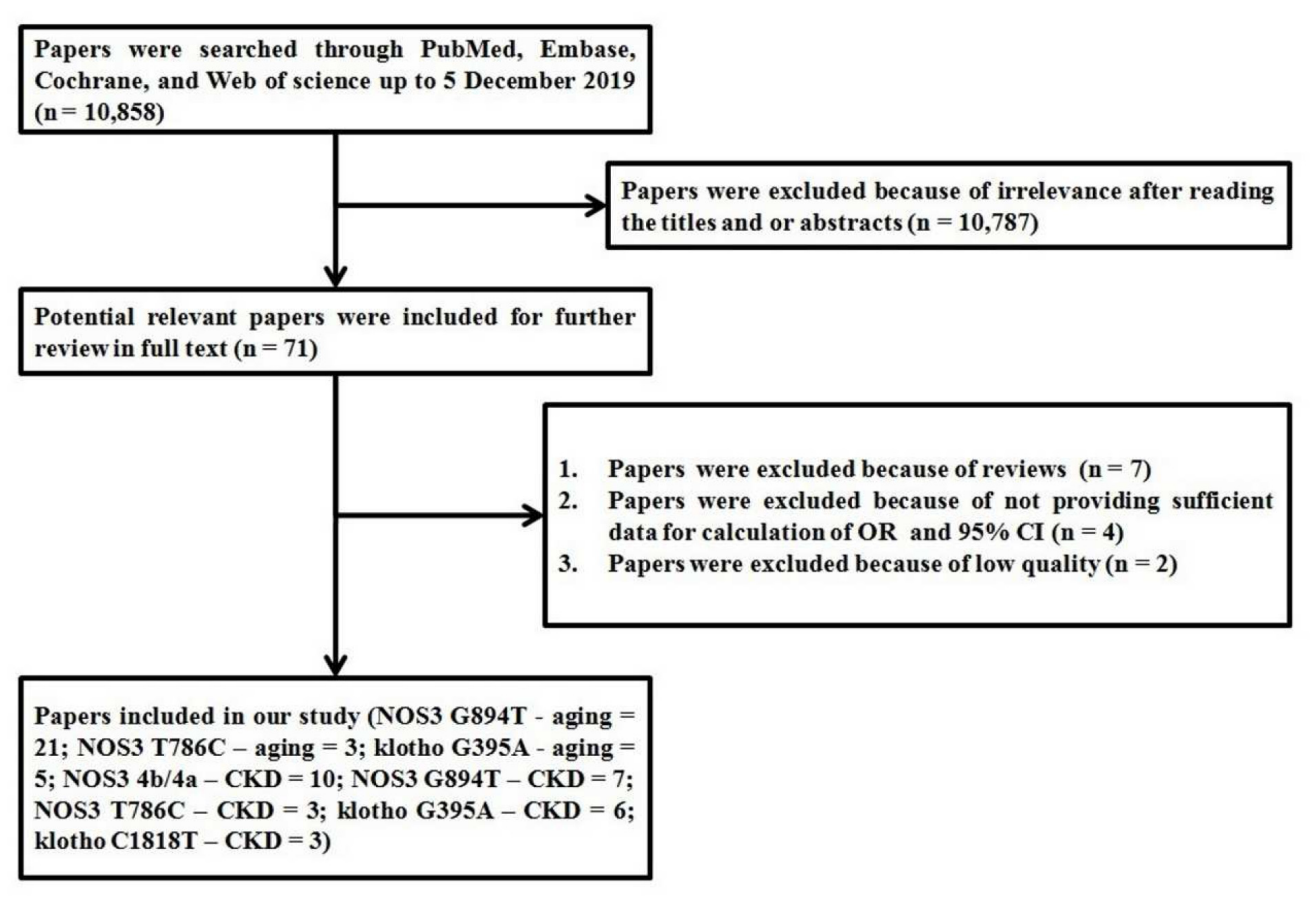
PRISMA flowchart of paper selection in our study.

Our final paper selection identified 21 papers ^22– 42^ assessing *NOS3* G894T gene polymorphisms in age-related cognitive impairment; three papers ^22, 43, 44^ assessing *NOS3* T786C gene polymorphisms in age-related cognitive impairment; five papers ^45– 49^ assessing *KL* G395A gene polymorphisms in age-related cognitive impairment; ten papers ^50– 59^ evaluating *NOS3* 4b/4a gene polymorphisms in CKD; seven papers ^55– 57, 59– 62^ evaluating *NOS3* G894T gene polymorphisms in CKD; three papers ^50, 55, 63^ assessing *NOS3* T786C gene polymorphisms in CKD; six papers ^64– 69^ assessing *KL* G395A gene polymorphisms in CKD; and three papers ^66– 68^ assessing *NOS3* C1818T gene polymorphisms in CKD. This number of papers were searched in PubMed, Embase, Cochrane, and Web of Science; and papers were selected in accordance with inclusion and exclusion criteria. In the initial searching, we identified 10,858 papers. Of those, 10,787 papers were excluded because of irrelevant topic. Moreover, 13 papers were also excluded because of the following reasons: review (seven), not providing required data for calculation of OR and 95%CI (four), and being of low-quality in accordance with NOS assessment (two). A flowchart describing eligibility pathway in our study is provided in Figure 1.

### Data synthesis

**Figure 2.**
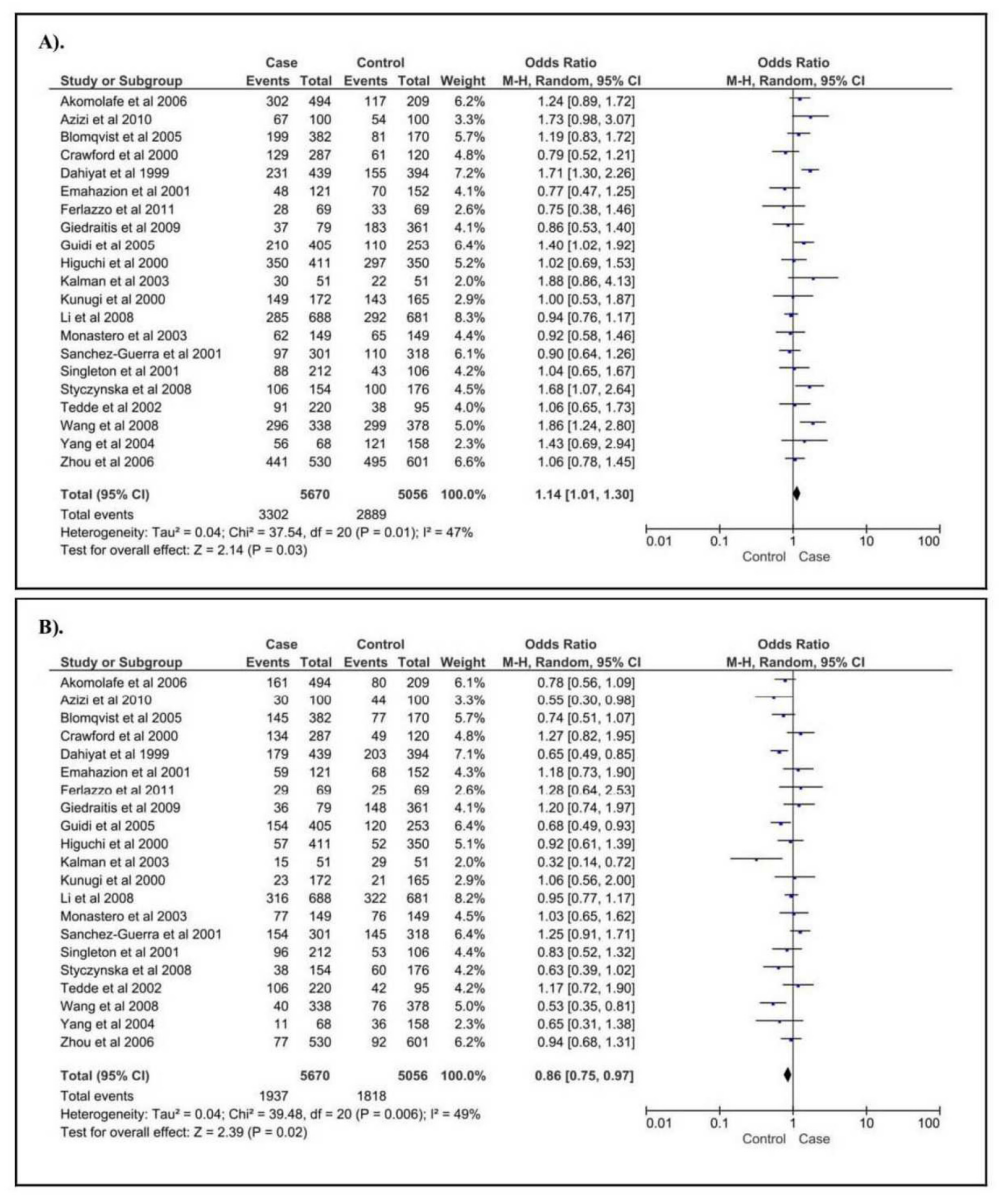
Forest plot of the association between NOS3 G894T gene polymorphism and the risk of age-related cognitive impairment. ( **A**). GG vs. GT+TT; ( **B**). GT vs. GG+TT.

For age-related cognitive impairment, we identified three SNPs available for meta-analysis calculation, including NOS3 G894T, NOS3 T786C, and KL G-395A. Of those, the correlation was only found in *NOS3* G894T gene variant. Conversely, we failed to clarify the correlation between the risk of age-related cognitive impairment and *NOS3* T786C and *KL* G395A gene polymorphism. For *NOS3* G894T, we found that increased risk of age-related cognitive impairment ( Figure 2A) was observed in GG genotype of *NOS3* G894T gene polymorphism (OR [95%CI] = 1.14 [1.01 - 1.30], p = 0.0320). On other hands, reduced risk of age-related cognitive impairment ( Figure 2B) was found in GT genotype of *NOS3* G894T gene variant (OR [95%CI] = 0.86 [0.75 - 0.97], p = 0.0170). The summary of the association between age-related cognitive impairment and the gene polymorphisms in *NOS3* and *KL* is given in Table 1.

**Table 1.** Summary of the association between the risk of age-related cognitive impairment and both *NOS3* and *KL* gene polymorphisms.

SNP	Allele & genotype	NS	Model	Value		OR	95%CI	pHet	pE	p-value
				Case (%)	Control (%)					
*NOS3* G894T	G vs. T	21	Random	75.3	75.1	1.08	0.99 - 1.18	0.0460	0.1210	0.0840
	T vs. G	21	Random	24.7	24.9	0.92	0.85 - 1.01	0.0460	0.1210	0.0840
	GG vs. GT+TT	21	Random	58.2	57.1	1.14	1.01 - 1.30	0.0100	0.1890	0.0320
	GT vs. GG+TT	21	Random	34.2	36.0	0.86	0.75 - 0.97	0.0060	0.2010	0.0170
	TT vs. GG+GT	21	Fixed	7.6	6.9	1.04	0.89 - 1.22	0.6770	<0.0001	0.6100
*NOS3* T786C	T vs. C	3	Fixed	75.7	80.2	0.93	0.81 - 1.07	0.6130	<0.0001	0.3010
	C vs. T	3	Fixed	24.3	19.8	1.08	0.94 - 1.24	0.6130	<0.0001	0.3010
	TT vs. TC+CC	3	Fixed	60.0	66.1	0.94	0.79 - 1.13	0.4960	<0.0001	0.5120
	TC vs. TT+CC	3	Fixed	31.3	28.1	1.00	0.84 - 1.19	0.5520	<0.0001	0.9980
	CC vs. TT+TC	3	Fixed	8.7	5.8	1.20	0.88 - 1.64	0.6970	<0.0001	0.2500
*KL *G395A	G vs. A	5	Random	84.6	84.7	0.93	0.73 - 1.18	0.0160	0.2210	0.5350
	A vs. G	5	Random	15.4	15.3	1.08	0.85 - 1.37	0.0160	0.2210	0.5350
	GG vs. GA+AA	5	Random	70.3	71.1	0.92	0.72 - 1.16	0.0450	0.2070	0.4730
	GA vs. GG+AA	5	Fixed	28.6	27.2	1.08	0.93 - 1.26	0.2520	0.1020	0.3060
	AA vs. GG+GA	3	Random	1.1	1.7	1.05	0.34 - 3.27	0.0280	0.8490	0.9270

SNP, single nucleotide polymorphism; NS, number of studies; OR, odd ratio; pHet, p heterogeneity; pE, p Egger.

**Figure 3.**
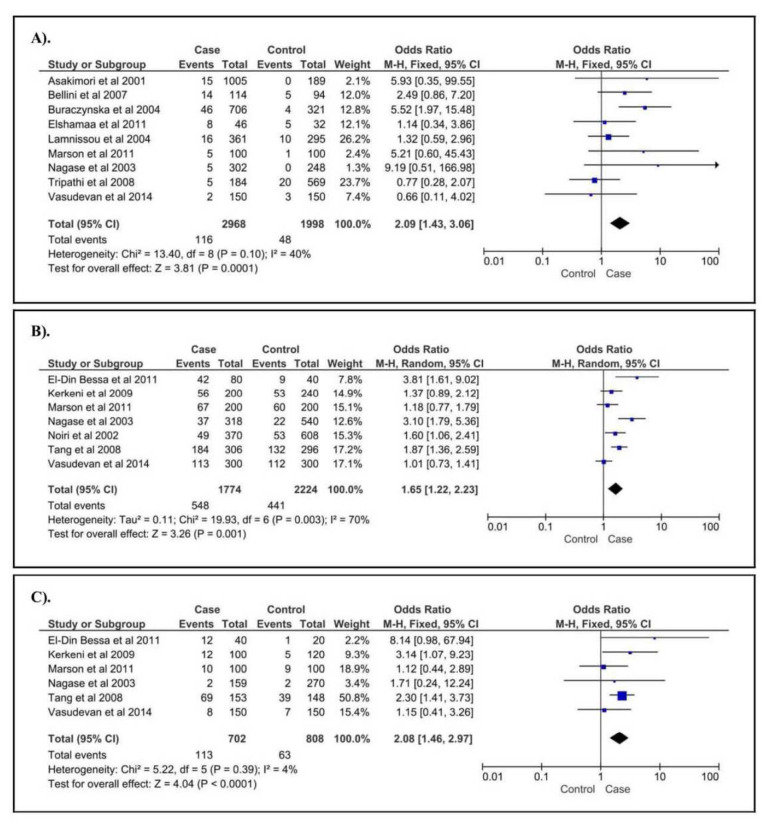
Forest plot of the association between *NOS3* gene polymorphism and the risk of CKD. **A**). 4a4a vs. 4b4b+4b4a of NOS3 4b/4a; **B**). T vs. G of NOS3 G-894T; **C**). TT vs. GG+GT of NOS3 G-894T.

For NOS gene polymorphisms in CKD patients, we identified three SNPs, *NOS3* 4b4a, *NOS3* G894T, and *NOS3* T786C. For *NOS3* 4b4a ( Figure 3A), we found that only the 4a4a genotype was associated with increased risk of CKD (OR [95%CI] = 2.09 [1.43 - 3.06], p < 0.0001). For *NOS3* G894T, we found that the T allele ( Figure 3B) and TT genotype ( Figure 3C) were, by 1.65 and 2.08-fold, respectively, associated with increased risk of CKD. Conversely, the G allele and GG genotype were associated to decreased risk of CKD. Moreover, for *NOS3* T786C, our findings failed to confirm the correlation in CKD patients.

**Figure 4.**
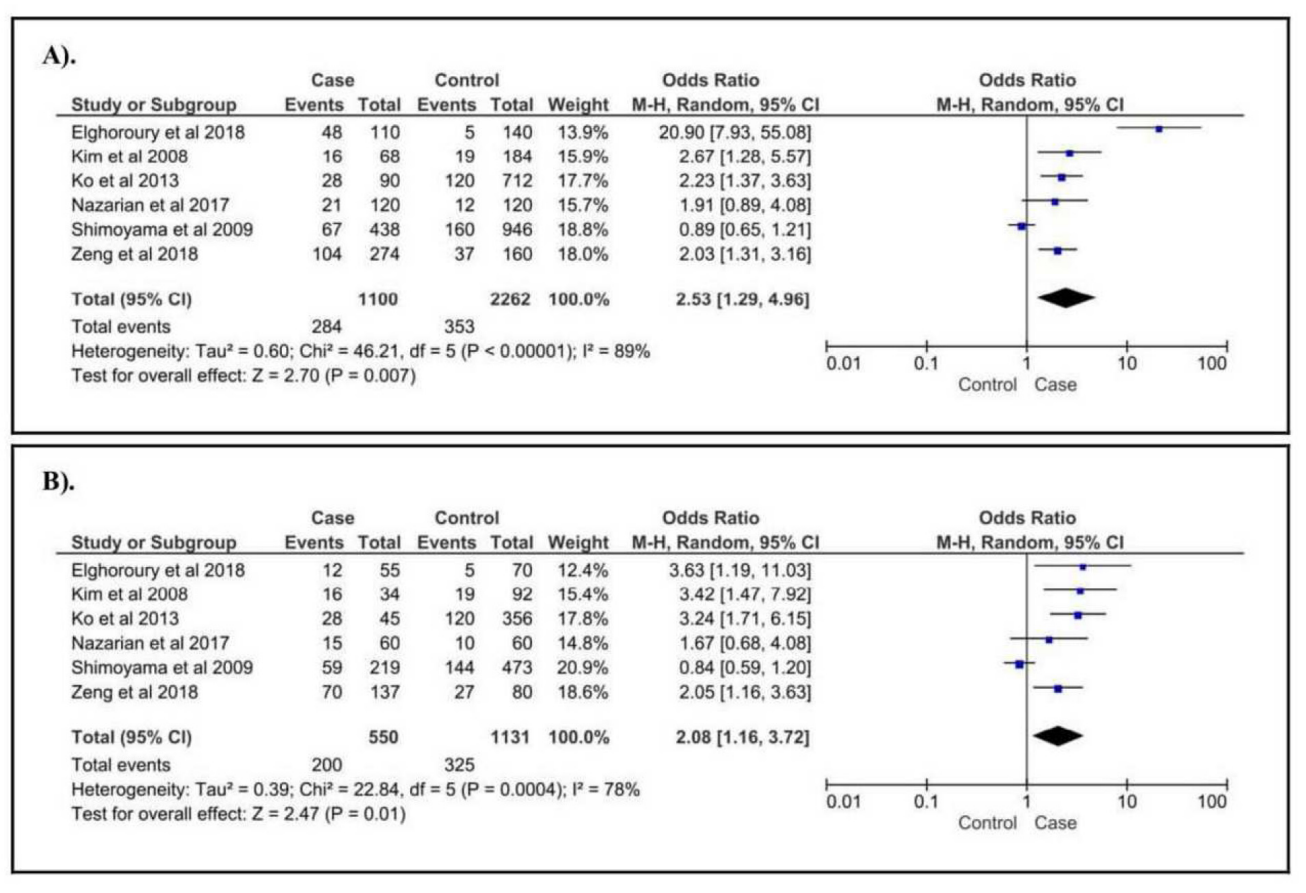
Forest plot of the association between *KL* G395A gene polymorphism and the risk of CKD. ( **A**). A vs. G of *KL* G-395A; ( **B**). GA vs. GG+AA of *KL* G395A.

Furthermore, for klotho gene polymorphisms in CKD patients, only two SNPs were compatible for our analysis, *KL* G-395A and *KL* C1818T. For *KL* G395A, we included six papers consisting of 550 cases and 1131 controls. Of those, G allele and GG genotype were observed having protective effect against CKD, and A allele ( Figure 4A) and GA genotype ( Figure 4B) were found susceptible for CKD. Moreover, for klotho C1818T, we failed to show the correlation in CKD patients. The summary of the correlation between the risk of CKD and the gene polymorphisms in *NOS3* and *KL* is described in Table 2.

**Table 2.** Summary of the association between the risk of CKD and both *NOS3* and *KL* gene polymorphisms.

SNP	Allele & genotype	NS	Model	Value		OR	95%CI	pHet	pE	p-value
				Case (%)	Control (%)					
*NOS3* 4b4a	4b vs. 4a	10	Random	83.5	86.4	0.80	0.60 - 1.07	<0.0001	0.4140	0.1300
	4a vs. 4b	10	Random	16.5	13.6	1.25	0.94 - 1.68	<0.0001	0.4140	0.1300
	4b4b vs. 4b4a+4a4a	10	Random	70.6	74.8	0.81	0.59 - 1.12	<0.0001	0.4490	0.2000
	4b4a vs. 4b4b+4a4a	10	Random	25.7	23.1	1.13	0.86 - 1.48	<0.0001	0.3610	0.3950
	4a4a vs. 4b4b+4b4a	9	Fixed	3.7	2.1	2.09	1.43 - 3.06	0.1170	0.5110	<0.0001
*NOS3* G894T	G vs. T	7	Random	69.1	80.2	0.61	0.45 - 0.82	0.0030	0.3310	0.0010
	T vs. G	7	Random	30.9	19.8	1.65	1.22 - 2.23	0.0030	0.3310	0.0010
	GG vs. GT+TT	7	Random	51.0	66.0	0.59	0.42 - 0.84	0.0160	0.3570	0.0030
	GT vs. GG+TT	7	Random	36.3	28.3	1.29	0.87 - 1.93	0.0020	0.4470	0.2070
	TT vs. GG+GT	6	Fixed	12.7	5.7	2.08	1.46 - 2.97	0.3930	0.1010	<0.0001
*NOS3* T786C	T vs. C	3	Random	76.4	73.5	0.80	0.45 - 1.45	0.0040	0.4720	0.4710
	C vs. T	3	Random	23.6	26.5	1.24	0.69 - 2.25	0.0040	0.4720	0.4710
	TT vs. TC+CC	3	Random	59.2	56.1	0.81	0.40 - 1.63	0.0090	0.5460	0.5570
	TC vs. TT+CC	3	Random	34.4	34.9	1.21	0.66 - 2.22	0.0290	0.4520	0.5340
	CC vs. TT+TC	3	Fixed	6.4	9.0	1.07	0.59 - 1.94	0.3240	0.2080	0.8280
*KL*G395A	G vs. A	6	Random	74.2	84.4	0.40	0.20 - 0.77	<0.0001	0.7720	0.0070
	A vs. G	6	Random	25.8	15.6	2.53	1.29 - 4.96	<0.0001	0.7720	0.0070
	GG vs. GA+AA	6	Random	56.0	70.0	0.36	0.17 - 0.76	<0.0001	0.8660	0.0070
	GA vs. GG+AA	6	Random	36.4	28.7	2.08	1.16 - 3.72	<0.0001	0.6230	0.0140
	AA vs. GG+GA	4	Random	7.6	1.2	2.96	0.84 - 10.42	0.0690	0.9400	0.0910
*KL* C1818T	C vs. T	3	Fixed	78.2	81.8	0.96	0.76 - 1.21	0.7020	<0.0001	0.7160
	T vs. C	3	Fixed	21.8	18.2	1.05	0.83 - 1.32	0.7020	<0.0001	0.7160
	CC vs. CT+TT	3	Fixed	59.3	65.4	0.96	0.72 - 1.27	0.7000	<0.0001	0.7600
	CT vs. CC+TT	3	Fixed	38.0	33.0	1.03	0.77 - 1.37	0.5620	<0.0001	0.8530
	TT vs. CC+T	2	Fixed	2.8	1.7	1.05	0.42 - 2.63	0.1800	1.0130	0.9130

SNP, single nucleotide polymorphism; NS, number of studies; OR, odd ratio; pHet, p heterogeneity; pE, p Egger.

### Source of heterogeneity

In the case of age-related cognitive impairment, for *NOS3* G894T gene polymorphism, except for TT genotype, the evidence for heterogeneity was found in all genetic models, and therefore we applied random effect model to analyze the data. For *NOS3* T786C, we found no evidence for heterogeneity, and therefore fixed effect model was used to analyze the data. For *KL* G395A, the evidence for heterogeneity was observed in all genetic models, except for the GA genotype. Therefore, we used random effect model to analyze the data. We provided the summary of heterogeneity analysis concerning this topic in Table 1.

In the case of CKD, for *NOS3* 4b/4a, evidence for heterogeneity was found in all genetic models, except for the 4a4a genotype. Therefore, we applied random effect model to analyze the data. Conversely, for the 4a4a genotype, because it was proven to have no heterogeneity, the analysis was performed using fixed effect model. Subsequently, for *NOS3* G894T, due to the lack of evidence for heterogeneity, we used a fixed effect model to analyze the TT genotype. On other hands, for other genetic models, a random effect model was applied to analyze the data. Moreover, for *NOS3* T786C, due to having the evidence for heterogeneity, we used a random effect model to analyze T and C alleles, the TT genotype, and the TC genotype. For the CC genotype, the association was determined using fixed effect model. Furthermore, for *KL* G395A, all genetic models were analyzed using a random effect model. For *KL* C1818T, a fixed effect model was used to analyze the correlation in all genetic models. We summarize the evidence of heterogeneity concerning the association between the risk of CKD and the gene polymorphisms of *NOS3* and *KL* in Table 2.

### Potential publication bias

We applied Egger's test to assess the potency of publication bias among studies. Our analysis revealed that, in the case of age-related cognitive impairment, the publication bias was found in TT genotype of *NOS3* G894T and all genetic models of *NOS3* T786C. Subsequently, in the case of CKD, we found that no publication bias was observed in all genetic models of *NOS3* 4b4a, *NOS3* G-894T, *NOS3* T-786C, and *KL* G-395A. However, publication bias was found in the C allele, T allele, CC genotype, and CT genotype of *KL* C1818T. The summary of Egger’s test in our study is presented in Table 1 for the case of age-related cognitive impairment and Table 2 for the case of CKD.

## Discussion

Our current study assessed the correlation between age-related cognitive impairment and the gene polymorphisms in *NOS3* ( *NOS3* G894T and *NOS3* T786C). Our results revealed that age-related cognitive impairment was not related to the gene polymorphism of *NOS3* T786C. On other hands, we found that the GG genotype was found to correlate with susceptibility to age-related cognitive impairment, and GT genotype was found to have a protective effect against age-related cognitive impairment. Our findings were consistent with those of a previous study ^70^. They also found that GG genotype of *NOS3* G894T was proven to associate with increased the susceptibility to age-related cognitive impairment. The evidence had confirmed that the polymorphism of *NOS3* G894T had been shown to correlate with NO basal production and NOS3 enzyme activity ^71^. Moreover, elevated NOS3 expression was also found to correlate with increased mitochondrial function in neurons ^72^. Therefore, it made sense that the *NOS3* G894T gene polymorphism was associated with age-related cognitive impairment as reported in our study. On other hands, we also reported the *NOS3* gene polymorphism in the case of CKD. Our results identified three SNPs available for the calculation of meta-analysis. However, the association with the risk of CKD was only observed in 4b/4a and G894T *NOS3* gene polymorphisms. For 4b/4a, our findings revealed that the 4a4a genotype was associated with increased risk of CKD. For the G894T gene polymorphism, we found that the T allele and TT genotype were observed to correlate with increased risk of CKD. Previous meta-analysis regarding this topic had been conducted ^73, 74^. However, our current study provided the update, and our current results were consistent with previous studies. Additionally, supporting our results, a previous study confirmed that the TT genotype and T allele of the G894T polymorphism, but not 4b/4a, were associated with the lower level of NO in circulation ^75^ and enzyme activity ^76^. Furthermore, lower NO levels compared to control was found in patients with CKD ^77^, although increased levels of NO were observed in CKD patients after dialysis ^78^. The similarity of the dominant role of the *NOS3* G894T gene polymorphism, both in age-related cognitive impairment and CKD, might explain the bridging mechanism between aging and CKD with NO involvement, in the context of gene-disease and gene-gene interactions.

The precise mechanism of NO in age-related cognitive impairment and CKD is undefined. However, some speculation may be proposed. Briefly, NO plays a significant role in cell growth and renal vasculature. It is widely known that NO plays as a vascular vasodilator. Additionally, NO may also inhibit the growth of mesangial cell and matrix production. The decreased level of NO in aging may cause to renal vasoconstriction, sodium retention, and increased matrix production and mesangial fibrosis ^79^. Moreover, NO isoforms are observed at higher levels in the medullary region than other regions. On other hands, in the renal cortex, the levels of NO isoforms are reduced. Therefore, they may contribute to the reduced perfusion of renal cortex in the elderly ^80^. The precise pathway of decreased level of NO in elderly remains confusing. However, several mechanisms have been proposed. First, oxidative stress is known to increase with age. It may stimulate to decrease the key factors for normal NO production, for example tetrahydrobiopterrin ^81^. Second, L-arginine is known to be key for the production of NO. The availability of this substrate may decline with advance age. While L-arginine is not an essential amino acid, a study had reported that the level of L-arginine was observed decreased in older rats. This indicates that L-arginine may play a crucial role as an essential amino acid in advance age, and therefore sufficient dietary intake may be required to maintain the NO production ^79^. Moreover, L-arginine level in circulation was also found to be significantly lower in patients with CKD than controls, and it was consistent with the level of NO ^77^. This suggested the pivotal role of L-arginine and NO in aging and CKD. Third, it is known that NOS is degraded by asymmetric dimethyl arginine (ADMA). Previous study in a rat model revealed that ADMA levels were observed higher in advance age. This suggests that elevated ADMA level may increase the degradation of NOS and cause lower NO production ^82^. Supporting this explanation, a study found that ADMA levels in circulation were higher in CKD patients than control, was contrary to the levels of NO and L-arginine ^77^. This explanation might bridge the mechanism between NO, aging, and CKD as reported in our present study.

While klotho was considered as one of the important mediators in aging, our findings failed to confirm the association between the *KL* G395A gene polymorphism and risk of age-related cognitive impairment. However, due to limited sample size, further investigation to assess this correlation was required. On other hands, correlating to CKD, our searching strategy identified *KL* G395A and C1818T as available for meta-analysis calculation. Our analysis confirmed that the association with CKD was only found in klotho G-395A gene polymorphism. We revealed that the A allele and GA genotype were correlated with increased risk of CKD. Until now, we have failed to obtain a systematic review or meta-analysis in the topic of either klotho in aging or in CKD. Therefore, a direct comprehensive comparison was unable to perform. However, it had been reported that α-klotho protein was related to the G395A polymorphism ^83^, and α-klotho protein in circulation was also proven by a large scale meta-analysis study to have positive correlation with renal function ^84^. It means that the lower level of klotho protein, the lower the renal function. Therefore, it might explain the results of our study confirming that the G395A gene polymorphism was correlated with the risk of CKD.

The theory explaining the exact mechanism between klotho, aging, and CKD is complicated and may involve genes, proteins, and target organ damage. At the genetic level, *KL* is expressed in limited tissues and cell types, and the highest expression is observed in distal convoluted tubules in the kidney and choroid plexus in the brain ^85^. Therefore, the klotho protein exists in two forms. One is the trans-membrane form expressed primarily in renal tubular cells, and the other is the secreted form circulating in the blood ^86^. Klotho protein level has been shown to correlate with human longevity ^87^. However, in advance age, the level of klotho protein is decreased ^88^, and this decreased level is associated with increased oxidative stress, proinflammatory cytokine production, and activation of endothelin signal transduction ^89^. Furthermore, the interaction between klotho and CKD is complex. It may involve specific signaling axis, defined as the klotho-fibroblast growth factor-23 (FGF23) signaling axis. Briefly, when the body has excessive amounts of phosphate, FGF23 is secreted. Subsequently, in the kidney, FGF23 may promote phosphate excretion into urine and suppress vitamin D synthesis. Consequently, it may induce negative phosphate balance. However, in this circumstance, FGF23 requires klotho to bind and activate FGF receptors. After FGF receptor activation by klotho, FGF23 binds to its receptor ^90^. There are four type of receptors for FGF23, such as FGFR1, FGFR2, FGFR3, and FGFR4. However, FGFR1 is the dominant receptor playing in this signaling pathway ^91^. When the binding between FGF23 and FGFR1 occurs, it may activate extracellular signals - regulated kinase (ERK) and serum/glucocorticoid-regulated kinase (SGK) signals. Furthermore, the phosphorylation of the Na ^+^/H ^+^ exchange regulatory cofactor (NHERF)-1 by SGK-1 was established to down-regulate membrane expression of sodium phosphate co-transporter NaPi-2a. Consequently, it may cause increasing urinary phosphate excretion ^92^. On other hands, the binding between FGF23 and FGFR1 may also suppress the expression of 1α-hydroxylase, the enzyme responsible for the production of 1.25(OH) _2_D. Therefore, it may participate to systemic mineral homeostasis and regulate the excretion of phosphate ^93^. In this context, if the level of klotho is decreased, it may lead to lower level of FGF23 and stimulate to hyperphosphatemia, one of the pathological states widely observed in CKD ^94^. Moreover, it was also reported that the level of klotho protein was found to decline and it was also accompanied by renal insufficiency in patients with CKD ^95^. Additionally, the gene-interaction studies also revealed that the decline of klotho level in subjects with CKD involved specific phenotypes, suggesting that klotho was independently involved in the pathogenesis of CKD ^85, 95^. This explanation might be a benchmark for the results of our study that klotho is an important mediator involved in the development of aging and CKD.

Our results have identified SNPs potentially involved in the pathogenesis of age-related cognitive impairment and CKD. Therefore, our current findings might help to elucidate the precise mechanism of aging and CKD in the perspective of clinical evidence and gene-disease interaction. Despite the limitations of our study, our findings might be as the initial step to develop further investigation for the management of aging and CKD. However, more studies on this topic are required to establish further due to some limitations, especially the wide context of aging that may make it difficult to conduct analysis and also may lead to high potency for bias.

In our current study, several limitations were noted. First, some factors that might influence NOS3 and klotho level including multiple sclerosis ^96^, asthma ^97^, chronic obstructive pulmonary disease ^98^, and cardiovascular disease ^5^ were not controlled for. Second, several potential confounding factors, mediators, and compensatory factors that might implicate the final findings of our study were not analyzed. Third, due to relatively small sample size, our findings should be interpreted with caution, considering the potency for bias. Fourth, most of study design in our included studies were cross-sectional. Thus, further studies with involving better study design might be required.

## Conclusion

Our present study has identified that *NOS3* G894T plays an important role in the pathogenesis of both age-related cognitive impairment and CKD. On other hand, while we have found an association between *KL* G395A gene polymorphism and the risk of CKD, its correlation with age-related cognitive impairment has not been clarified. Our current study may contribute to better understanding regarding the role of *NOS3* and *KL* in the pathogenesis of age-related cognitive impairment and CKD.

## Data availability

### Underlying data

All data underlying the results are available as part of the article and no additional source data are required.

### Reporting guidelines

Figshare: PRISMA checklist for ‘Nitride oxide synthase 3 and klotho gene polymorphisms in the pathogenesis of chronic kidney disease and age-related cognitive impairment: a systematic review and meta-analysis’. https://doi.org/10.6084/m9.figshare.12016782 ^20^.
