# Supplementary material for: Nitride oxide synthase 3 and klotho gene polymorphisms in the pathogenesis of chronic kidney disease and age-related cognitive impairment: a systematic review and meta-analysis
Source: F1000Res. 2021 Mar 19;9:252. Originally published 2020 Apr 9. [Version 2] doi: 10.12688/f1000research.22989.2 (PMC8112466; doi:10.12688/f1000research.22989.2)
Supplement: Peer review history

# Peer review: Reviewer response for version 2

**DOI:** 10.5256/f1000research.55513.r81755

**Published:** 2021-05-10

**Authors:** Sang Won Park

**Affiliations:** 1 Department of Pharmacology, College of Medicine, Institute of Health Sciences, Gyeongsang National University, Jinju, South Korea

The reviewer’s concerns have been addressed in the response and the last sentence (“We only tried to identify….gene-gene interaction.”) should be included in the manuscript to avoid the readers misunderstood.

Are the rationale for, and objectives of, the Systematic Review clearly stated?

Partly

Is the statistical analysis and its interpretation appropriate?

Partly

Are sufficient details of the methods and analysis provided to allow replication by others?

Partly

Are the conclusions drawn adequately supported by the results presented in the review?

Partly

Reviewer Expertise:

Acute kidney injury, preclinical study,

I confirm that I have read this submission and believe that I have an appropriate level of expertise to confirm that it is of an acceptable scientific standard.

# Peer review: Reviewer response for version 2

**DOI:** 10.5256/f1000research.55513.r81754

**Published:** 2021-03-22

**Authors:** Hongliang Zhang

**Affiliations:** 1 Department of Life Sciences, National Natural Science Foundation of China, Beijing, China

The reviewer's concerns have been adequately addressed.

Are the rationale for, and objectives of, the Systematic Review clearly stated?

No

Is the statistical analysis and its interpretation appropriate?

I cannot comment. A qualified statistician is required.

Are sufficient details of the methods and analysis provided to allow replication by others?

Partly

Are the conclusions drawn adequately supported by the results presented in the review?

No

Reviewer Expertise:

nephrology, neurology, neuroimmunology, neuroimaging, neuroscience

I confirm that I have read this submission and believe that I have an appropriate level of expertise to confirm that it is of an acceptable scientific standard.

# Response

**Published:** 2021-04-19

**Authors:** Jonny Fajar

**Affiliations:** Brawijaya Internal Medicine Research Center, Department of Internal Medicine, Faculty of Medicine, Universitas Brawijaya, Malang, Indonesia, Indonesia

We are very grateful for the invaluable advice.

# Peer review: Reviewer response for version 1

**DOI:** 10.5256/f1000research.25378.r68104

**Published:** 2020-08-10

**Authors:** Sang Won Park

**Affiliations:** 1 Department of Pharmacology, College of Medicine, Institute of Health Sciences, Gyeongsang National University, Jinju, South Korea

This study performed a meta-analysis for an association study of NOS3 and klotho polymorphisms with CKD and age-related cognitive impairment (ACI). Several NOS3 and klotho polymorphic nucleotide sites were analyzed in 48 selected papers. The correlation was found only in NOS3 G894T with ACI. On the other hand, the correlation was found in NOS 4b4a, NOS3 G894T, and KL G395A with CKD. The authors conclude that NOS3 G894T has a crucial role in the pathogenesis of both CKD and ACI. I have a major concern as follows.

 The purpose of this paper is not clear. Which is to reveal common polymorphisms in ACI and CKD to associate these two pathologies, to find polymorphisms correlated in CKD and aging, by using ACI as a marker of aging, or other purposes? However, ACI is not a good marker of aging in a meta-analysis because ACI has pathological complexity. According to the purpose, the parameter selection of polymorphic genes should be different. NOS3 functions in the cardiovascular system and the NOS3 polymorphisms are susceptible to diseases, such as hypertension, atherosclerosis, stroke, and other complications. Klotho functions in the endocrine FGF-mediated metabolic processes, such as regulating insulin secretion, feeding, and renal reabsorption of calcium and phosphate; therefore, it is associated with diabetes, CKD, and other metabolic disorders. In this regard, a meta-analysis to reveal the correlation between NOS3 and ACI should be performed separately to the meta-analysis to reveal the correlation between Klotho with CKD. Without a clear purpose, correlation studies of NOS3 and Klotho in both ACI and CKD are not clinically important. Please clarify your purpose of the study and revise your introduction and conclusions with appropriate references. You may be required additional meta-analysis on other gene polymorphisms related to ACI and CKD.

Are the rationale for, and objectives of, the Systematic Review clearly stated?

Partly

Is the statistical analysis and its interpretation appropriate?

Partly

Are sufficient details of the methods and analysis provided to allow replication by others?

Partly

Are the conclusions drawn adequately supported by the results presented in the review?

Partly

Reviewer Expertise:

Acute kidney injury, preclinical study,

I confirm that I have read this submission and believe that I have an appropriate level of expertise to confirm that it is of an acceptable scientific standard, however I have significant reservations, as outlined above.

# Response

**Published:** 2021-03-16

**Authors:** Jonny Fajar

**Affiliations:** Brawijaya Internal Medicine Research Center, Department of Internal Medicine, Faculty of Medicine, Universitas Brawijaya, Malang, Indonesia, Indonesia

Our study aimed to perform a meta-analysis concerning the role of NOS and klotho gene polymorphisms in the case of age-related cognitive impairment and CKD. In our paper, we only tried to identify the potential SNPs having the role in the development of CKD and the development of age related cognitive impairment. We did not propose the possible causal correlation between age related cognitive impairment and CKD. They are two distinct condition. We only tried to identify the similar SNPs between CKD and age related to cognitive impairment, and in the future, this similarity might be used in the concept of gene-gene interaction.

# Peer review: Reviewer response for version 1

**DOI:** 10.5256/f1000research.25378.r66026

**Published:** 2020-07-06

**Authors:** Hongliang Zhang

**Affiliations:** 1 Department of Life Sciences, National Natural Science Foundation of China, Beijing, China

The authors performed a meta-analysis to assess the role of NOS and klotho single nucleotide polymorphisms (SNPs) in the pathogenesis of CKD and age-related cognitive impairment. They identified SNPs potentially involved in the pathogenesis of age-related cognitive impairment and CKD. They concluded that *NOS3 *G894T gene polymorphism has a crucial role in the pathogenesis of both CKD and age-related cognitive impairment. Overall, the study is well designed and conducted. The PRISMA checklist was well implemented.

**Major concern:**

1. A hypothesis is missing and validation is lacking. Association does not necessarily mean a causal relationship. Confounding factors, mediators, and compensatory factors can be implicated in the role of NOS and klotho single nucleotide polymorphisms (SNPs) in the pathogenesis of CKD and age-related cognitive impairment.
2. The definition and diagnostic criteria seem lacking for age-related cognitive impairment. Of note is that age-related cognitive impairment is not a disease. Ref 13 fails to give a definition.

**Minor points:**

1. " and being of low-quality (two)." in the paper selection should be specified.
2. "a candle in the darkness" is kind of exaggerated. It is simply a meta-analysis. The data should not be over-interpreted.
3. The language should be polished. "frightening" appears frightening in scientific writings. " 13 papers were also excluded because of review(s)" should be rephrased. Please use "we found that...... " instead of "our results found that...... ".
4. "For age-related cognitive impairment, we identified three SNPs available for meta-analysis calculation, such as: *NOS3 *G894T, *NOS3 *T786C, and *KL *G-395A." can be rephrased to "For age-related cognitive impairment, we identified three SNPs available for meta-analysis calculation, including *NOS3 *G894T, *NOS3 *T786C, and *KL *G-395A."

Are the rationale for, and objectives of, the Systematic Review clearly stated?

No

Is the statistical analysis and its interpretation appropriate?

I cannot comment. A qualified statistician is required.

Are sufficient details of the methods and analysis provided to allow replication by others?

Partly

Are the conclusions drawn adequately supported by the results presented in the review?

No

Reviewer Expertise:

nephrology, neurology, neuroimmunology, neuroimaging, neuroscience

I confirm that I have read this submission and believe that I have an appropriate level of expertise to confirm that it is of an acceptable scientific standard, however I have significant reservations, as outlined above.

# Response

**Published:** 2021-03-16

**Authors:** Jonny Fajar

**Affiliations:** Brawijaya Internal Medicine Research Center, Department of Internal Medicine, Faculty of Medicine, Universitas Brawijaya, Malang, Indonesia, Indonesia

1. A hypothesis is missing and validation is lacking. Association does not necessarily mean a causal relationship. Confounding factors, mediators, and compensatory factors can be implicated in the role of NOS and klotho single nucleotide polymorphisms (SNPs) in the pathogenesis of CKD and age-related cognitive impairment.

 Response: the potential confounding factors, mediators, and compensatory factors that might affect the final findings of our study have been provided in the limitations.  

 2. The definition and diagnostic criteria seem lacking for age-related cognitive impairment. Of note is that age-related cognitive impairment is not a disease. Ref 13 fails to give a definition.

 Response: In the second paragraph of introduction, we did not provide the definition and diagnostic criteria of age-related cognitive impairment. We only described the problems in the context of aging and age related cognitive impairment.

 3. " and being of low-quality (two)." in the paper selection should be specified.

 Response: We have revised the sentence.

 4. "a candle in the darkness" is kind of exaggerated. It is simply a meta-analysis. The data should not be over-interpreted.

 Response: We have revised the sentence.

 5. The language should be polished. "frightening" appears frightening in scientific writings. " 13 papers were also excluded because of review(s)" should be rephrased. Please use "we found that...... " instead of "our results found that...... ".

 Response: We have revised the sentence.

 6. "For age-related cognitive impairment, we identified three SNPs available for meta-analysis calculation, such as: NOS3 G894T, NOS3 T786C, and KL G-395A." can be rephrased to "For age-related cognitive impairment, we identified three SNPs available for meta-analysis calculation, including NOS3 G894T, NOS3 T786C, and KL G-395A."

 Response: We have revised the sentence.
